# Postoperative delayed gastric emptying: may gut microbiota play a role?

**DOI:** 10.3389/fcimb.2024.1449530

**Published:** 2024-08-13

**Authors:** Zhiyi Wang, Chuanbo Liu, Kaiwen Hu, Minghuan Zuo, Zhen Tian, Yue Wei, Qin Zhou, Quanwang Li

**Affiliations:** ^1^ Graduate School of Beijing, University of Chinese Medicine, Beijing, China; ^2^ Department of Oncology, Dongfang Hospital, Beijing University of Chinese Medicine, Beijing, China

**Keywords:** postoperative delayed gastric emptying, gut microbiota, postoperative complications, gastric emptying, gastroparesis

## Abstract

Postoperative delayed gastric emptying is a prevalent complication following surgical procedures, imposing heavy physical and financial burdens on patients. However, current treatment options remain suboptimal. In recent years, an increasing number of studies have highlighted that the gut microbiota and its metabolites are closely associated with postoperative complications. Various factors can disrupt the gut microbiome after surgery. This review discusses the potential mechanisms by which the gut microbiota and their metabolites may contribute to the pathogenesis of postoperative delayed gastric emptying. However, the current knowledge base is limited in terms of fully understanding the exact mechanisms involved. It is therefore evident that further research is required to fully elucidate the role of the gut microbiome in postoperative delayed gastric emptying, with the aim of uncovering new possibilities for preventive measures and therapeutic treatments.

## 1 Introduction

Postoperative delayed gastric emptying (DGE), also known as postoperative gastroparesis syndrome, is defined as delayed gastric emptying in the absence of mechanical obstruction, which is clinically manifested by nausea, vomiting, early satiety, postprandial fullness, and epigastric pain (Camilleri and Sanders, 2022; Camilleri et al., 2022). It is a common complication following gastrointestinal surgery (Eshuis et al., 2015; Takatsuki et al., 2021; Baia et al., 2022) and can also manifest after non-abdominal procedures, with varying incidence among different surgical specialties. Statistically, approximately 7%-59% of patients may experience DGE after pancreaticoduodenectomy (Parmar et al., 2013; Mirrielees et al., 2020; Li et al., 2021), while the estimated incidence after gastrectomy ranges from 6% to 23% (Zhu et al., 2018). Postoperative DGE can increase the length of stay (Smits et al., 2022), reduce the quality of life, increase the economic burden, and may have a negative impact on oncological outcomes (Futagawa et al., 2017; Dominguez et al., 2023). Restoring gastrointestinal function is crucial for rapid recovery in postoperative patients. Unfortunately, there is currently a lack of ideal clinical therapeutic options for postoperative DGE, emphasizing the desperate necessity for the development of safer and more efficient treatments.

The pathogenesis of postoperative DGE remains poorly deconvoluted. Currently, it is thought to be multifactorial, involving nerve damage, gastric smooth muscle lesions, interstitial cells of Cajal (ICCs) damage, and other unknown elements. Recently, there has been an emerging awareness that the gut microbiota and their metabolites may be significant conductors in postoperative recovery (Liu et al., 2023; Yu et al., 2023; Zhang et al., 2023). It has been observed that gastrointestinal procedures can change the composition and structure of the intestinal flora, leading to postoperative intestinal microbial dysbiosis (Guyton and Alverdy, 2017), which may contribute to the development of postoperative gastrointestinal complications (Li et al., 2020; Gibiino et al., 2022; Zheng et al., 2023; Zhang et al., 2024). An early study in 1967 showed that germ-free mice had significantly lower gastric emptying and intestinal transit rates compared to conventional mice, suggesting a significant relationship between intestinal flora and gastrointestinal motility (Abrams and Bishop, 1967; Waclawiková et al., 2022). In addition, differences in the structure of the intestinal flora between Wistar rats, which are characterized by gastroparesis, and SD rats have been observed (Dalziel et al., 2017). Moreover, it is reported that duodenal flora is associated with DGE (Shanahan et al., 2023). These results suggest that dysbiosis of the gut microbiome may be closely related to the pathogenesis of DGE (Mandarino et al., 2023). Previous literature investigated the mechanisms of intestinal flora and their metabolites in postoperative ileus (Ma et al., 2023). Although postoperative acute gastroparesis may be a part of the ileus syndrome, dysmotility may not manifest throughout the gastrointestinal tract due to the diversity between regions of the alimentary tract (Sanders et al., 2012). The present article focuses on the potential pathogenic mechanisms of intestinal flora in postoperative DGE, trying to inspire viable and rational therapeutic strategies.

## 2 Physiology of gastric emptying and general pathogenesis of postoperative delayed gastric emptying

Gastric emptying and its regulation involve a complex series of sequential processes that encompass the central nervous system (CNS), the autonomic nervous system (ANS), the enteric nervous system (ENS), smooth muscle cells, ICCs, and gastrointestinal hormones, among others (Liu et al., 2021b). Gastric motility is primarily regulated by extrinsic innervation from the sympathetic and parasympathetic nervous systems, with intrinsic innervation providing local control. Current understanding suggests that sympathetic nerves have a limited role in regulating gastrointestinal motility, while parasympathetic nerves (especially the vagus nerves) dominating excitatory effects on gastrointestinal motility. In addition, endocrine regulation, which is mainly achieved by gastrointestinal hormones, is another significant form of regulation (Sanger and Lee, 2008). These hormones, also known as brain-gut peptides, dominantly including gastrin, motilin, Ghrelin, cholecystokinin, vasoactive intestinal peptide, glucagon-like peptide-1 (GLP-1), etc. Among them, ghrelin and motilin agonists are being investigated as potential targets for the treatment of gastroparesis (Sanger and Furness, 2016). Any dysfunction of these components can result in gastric motility disorders.

The research dilemma in postoperative DGE is partly due to the unclear pathogenesis, necessitating further exploration. Current understanding suggests that DGE is a neuromuscular dysfunction disorder potentially linked to various factors, such as autonomic and enteric neuropathy, smooth muscle dysfunction, abnormal duodenal antral coordination, gastric dysrhythmia, gastrointestinal hormones imbalances, etc (Sullivan et al., 2020; Abell et al., 2021; Gilbert et al., 2023). Firstly, surgical procedures may lead to anatomic modifications that can cause regional abnormalities in the motility patterns of the fundus, body, antrum, and pylorus, ultimately resulting in DGE. Additionally, intraoperative damage to the vagus nerve is a significant factor in postoperative DGE (Ye et al., 2022). Also, surgical stress can lead to sympathetic activation and decreased parasympathetic excitability, further contributing to the slowing of gastric emptying (Nguyen et al., 2020). On the other hand, ENS is essential for coordinating gastrointestinal motility. Reduced density of intestinal neurons in the gastric mucosa has been demonstrated in patients with gastroparesis and in animal models (Kedar et al., 2011; Baker et al., 2020). Gastrointestinal hormones are involved in the regulation of gastrointestinal motility after surgery (Stengel and Taché, 2014). Research has shown that perioperative serum levels of gastrin, motilin, and substance P are positively associated with early flatulence, while negatively correlated with cholecystokinin and somatostatin levels (Chen et al., 2022). There is ample evidence that loss or structural abnormalities of ICCs are implicated in the pathogenesis of delayed gastric emptying (Grover et al., 2019; Liu et al., 2021a). In patients with gastroparesis, a reduction in the number of ICCs in biopsy tissue specimens from the jejunum, colon, and antrum has been reported (Chikkamenahalli et al., 2020). Besides, ischemic injury-induced cell damage is recognized as a mechanism underlying postoperative gastroparesis (Vélez and Kuo, 2021).

## 3 Various factors influence intestinal microbiota after surgery

Intestinal microbiota refers to the microorganisms that colonize and survive in the gastrointestinal tract in a complex and delicate balance, including trillions of bacteria, archaea, fungi, protozoa, and viruses, with bacteria being the most prevalent (D’Argenio and Salvatore, 2015). Intestinal bacteria consists of nine phyla, of which *Bacillota* and *Bacteroidota* are the dominant ones. Emerging delect studies have demonstrated that surgical interventions can significantly shift the abundance and function of the gut flora and their metabolites (Guyton and Alverdy, 2017; Shao et al., 2017; Koliarakis et al., 2020; Gibiino et al., 2022). In 2021, a systematic review encompassing 14 studies for the first time highlighted substantial alterations in the gut microbiome following surgical procedures (Ferrie et al., 2021). Subsequently, another meta-analysis, which included 33 gastrointestinal surgeries, further confirmed the alterations in gut microbiome composition following surgical procedures (Tarazi et al., 2022).

The alterations in the structure and function of the gut microbiota postsurgery are influenced by various factors, such as the type of operation and the methods of digestive tract reconstruction (Guyton and Alverdy, 2017; Yao et al., 2022; Tsigalou et al., 2023). For instance, patients with gastric cancer who underwent radical distal gastrectomy exhibited a notable rise in *Escherichia/Shigella*, *Akkermansia*, *Dialister*, *and Prevotella*, while a significant decline in bacteria such as *Klebsiella*, *Streptococcus*, *Phascolarctobacterium*, and *Bifidobacterium* after surgery compared to the preoperative period (Liang et al., 2019). Another study showed that the diversity and abundance of the gut microbiota in gastric cancer patients after gastrectomy were higher than in the normal control group, with elevated levels of oral microorganisms, aerobic bacteria, and facultative anaerobes (Erawijantari et al., 2020). Gastrectomy leads to a reduction in gastric acid secretion and damage to the gastric mucosal barrier, allowing oral bacteria to survive and eventually colonize the intestine (Maksimaityte et al., 2021). A pilot study found that the relative abundance of *Pseudomonadota* in colorectal cancer patients after surgery was significantly higher in comparison to pre-surgery levels. Additionally, the genus *Klebsiella* exhibited a higher proportion than that observed prior to surgery (Cong et al., 2018). A case-control study of patients with colorectal cancer revealed significant alterations in the gut microbiota following surgery, including a decrease in obligate anaerobes, an increase in pathogenic bacteria, and a reduction in short-chain fatty acids (Ohigashi et al., 2013). In a recent study, Fang and colleagues revealed a significant increase in the prevalence of *Klebsiella* and *Lachnoclostridium* in the intestinal microbiota of patients after pancreaticoduodenectomy (Fang et al., 2024). It is evident that there is no consensus regarding the impact of surgical procedures on the microbiological structure of the patient’s gut. **Table 1** provides a summary of several studies on the changes in the gut microbiota in patients following different types of surgery.

**Table 1:** The impact of different surgery types on the gut microbiota.

Surgery type	Patients	Sample type	Methods	Microbiota changes	Reference
radical distal gastrectomy	advanced gastric adenocarcinoma	facal samples	16S rRNA gene sequencing	↑ *Akkermansia, Escherichia/Shigella, Dialister, Prevotella*; ↓ *Klebsiella*, *Streptococcus*, *Phascolarctobacterium*, *Bifidobacterium*	Liang et al., 2019
gastrectomy	gastric cancer	facal samples	shotgun metagenomics sequencing	↑ *Streptococcus*, *Enterococcus*, *Escherichia*, *Enterobacter*	Erawijantari et al., 2020
palliative surgery or radical surgery for colorectal cancer	colorectal cancer	facal samples	16S rRNA gene sequencing and real-time quantitative PCR	↑ *Klebsiella*, *Proteobacteria*	Cong et al., 2018
colorectal cancer surgery	colorectal cancer	facal samples	16S rRNA-targeted reverse transcription-quantitative PCR	↓ obligate anaerobes; ↑ *Enterobacteriaceae*, *Enterococcus*, *Staphylococcus*, *Pseudomonas*	Ohigashi et al., 2013
pancreaticoduodenectomy and gastrojejunal anastomotic	periampullary carcinoma	facal samples	16S rDNA gene sequencing	↑ *Klebsiella*, *Lachnoclostridium*; ↓ *Bifidobacterium*, *Prevotella*	Fang et al., 2024

For another, perioperative medications could be a significant cause of changes in the composition and abundance of the gut microbiota (Liufu et al., 2020). Surgical bowel preparations, such as intestinal mechanical preparations, and oral or intravenous antibiotics, can significantly impact the gut microbiota composition following surgery (Omar Al-Hassi et al., 2018; Nalluri-Butz et al., 2022). As evidenced by a 31-fold decrease in total microbial load after the bowel cleaning. However, this change was mostly recovered within a period of 14 days (Jalanka et al., 2015). Multiple studies have demonstrated that antibiotics, commonly prescribed in the perioperative period to prevent infections, are capable of disrupting the balance of the intestinal microflora by reducing the abundance and diversity of microbiota (Duan et al., 2022; Xue et al., 2023). Moreover, intraoperative fluid therapy regimens and postoperative application of proton pump inhibitors can also alter the perioperative gut flora characteristics of patients (Jackson et al., 2016; Lu et al., 2022). Interestingly, postoperative use of proton pump inhibitors (PPIs) has been linked to a significantly higher incidence of delayed gastric emptying after pancreaticoduodenectomy (Panni et al., 2023). But it is unclear to what extent the gut microbiota contributes to the adverse effects associated with PPIs use (Macke et al., 2020). Exposure to anesthetics can lead to significant and lasting changes in gut microbiome diversity (Serbanescu et al., 2019; Wang et al., 2019; Minerbi and Shen, 2022). Opioid medications are the mainstay for controlling perioperative pain. Treatment with opioids, such as morphine, has been shown to have selective effects on the gut microbiome, primarily leading to an increase in potentially harmful bacteria and a decrease in potentially beneficial bacteria (Taboun and Sadeghi, 2023).

**Figure 1:**
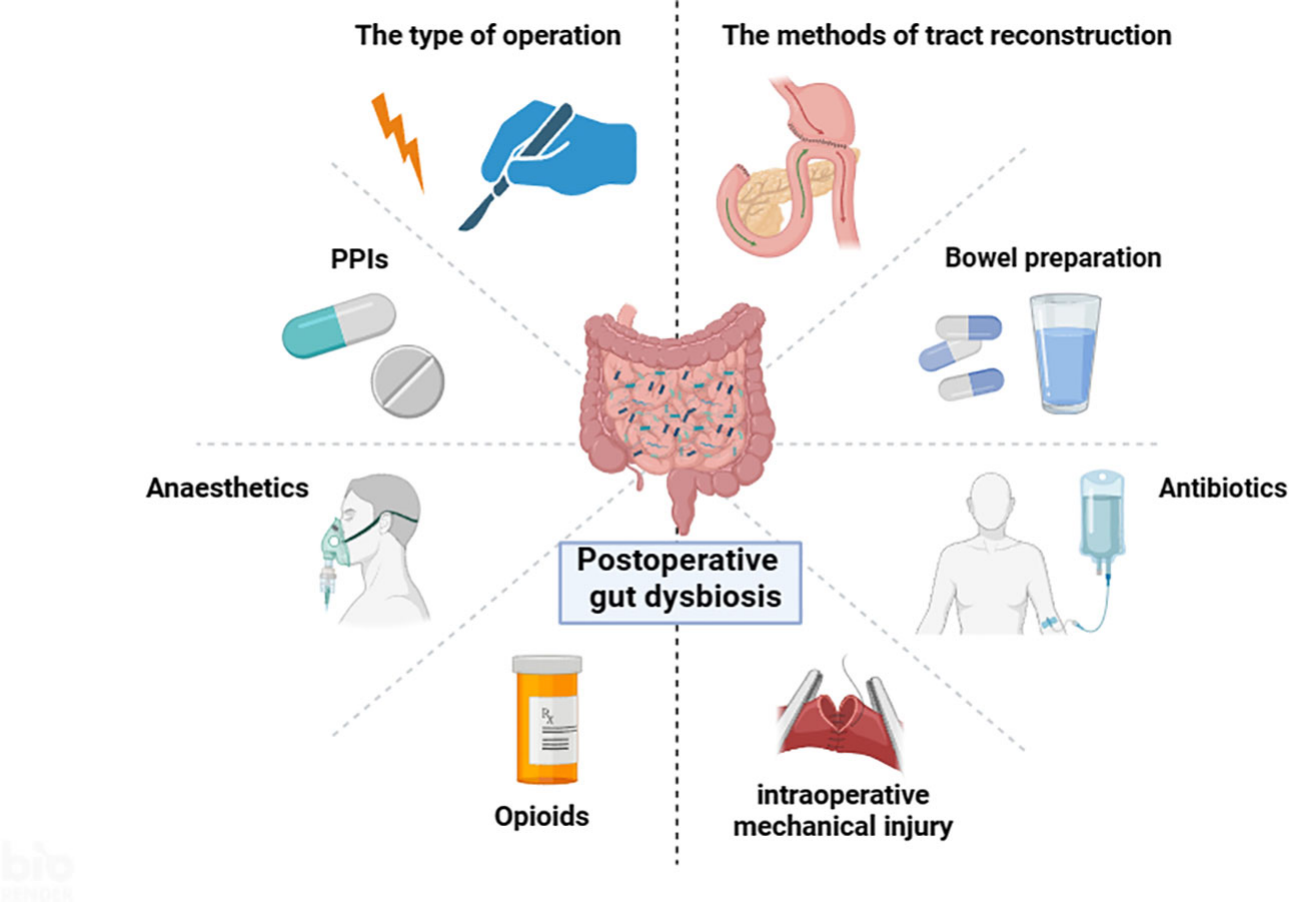
Multiple factors influence the intestinal microbiota during the perioperative period (By BioRender.com).

Besides, intraoperative mechanical injury is another important factor that affects the intestinal flora. Gastric venous stasis can lead to gastric ischemia and impaired gastric emptying (Loos et al., 2022; Stoop et al., 2023). Preserving the correct gastric vasculature may help reduce gastric venous congestion and ischemia, thereby maintaining gastric motility (Marchegiani et al., 2023). Intraoperative ischemia, hypoxia, and ischemia-reperfusion injury can induce changes in the intestinal flora (Li et al., 2022). The intestinal mucosa is particularly sensitive to ischemia and hypoxia. Tian et al. observed that systemic hypovolemia-induced intestinal ischemia-reperfusion injury disturbed the intestinal microbiome in mice, resulting in a decrease in *Bacteroidota* and an increase in *Pseudomonadota* (Tian et al., 2016). Collectively, a growing body of evidence has demonstrated that manifold confounders during the perioperative period may contribute to the changes of the gut microbiota (**Figure 1**). However, these changes are quite heterogeneous across different studies.

## 4 Potential abnormal gastrointestinal microbiota associated with delayed gastric emptying

In recent years, clinical and fundamental scientific studies have highlighted the role of gut microbiota and their metabolites in postoperative complications (Schmitt et al., 2019; Lederer et al., 2021). For instance, in a prospective cohort study conducted by Shogan and colleagues, which included 101 patients undergoing colorectal surgery, analysis of fecal samples collected on the day of surgery and postoperative day 2 showed an increase in the abundance of *Bacteroidota*, *Parabacteroides*, and *Ruminococcus* in patients who developed postoperative intestinal obstruction (Shogan et al., 2020). This suggests that alterations in the intestinal flora may be a contributing factor in the development of postoperative intestinal obstruction. Nevertheless, few studies have directly examined the gut microbiota changes in postoperative DGE to address the connection between postoperative DGE and gut flora characteristics. An analysis of the intestinal flora in 14 patients who underwent gastrectomy and Billroth II reconstruction for gastric cancer revealed a notable decrease in both α-diversity and β-diversity of the intestinal flora, and these changes in intestinal microorganisms were closely associated with gastrointestinal symptoms (Bausys et al., 2022). The duodenum has been identified as a crucial component in the pathogenesis of upper gastrointestinal diseases. Shanahan et al. conducted an analysis of the mucosa-associated flora through duodenal biopsy. They demonstrated a negative correlation between the relative abundance of *Veillonella* spp. and gastric emptying time (Shanahan et al., 2023). Lung transplant recipients with DGE have reduced microbial diversity in gastric fluid compared to those with normal gastric emptying (Lötstedt et al., 2021). These findings suggest that gut microorganisms may play a potential role in the initiation and progression of gastroparesis (Mandarino et al., 2023), which warrants further investigation. It is noteworthy that most current studies are based on samples of gut flora from feces, while the small bowel and mucosal microbes may need more focus.

## 5 Possible mechanisms of gut dysbiosis affecting postoperative gastric emptying

**Figure 2:**
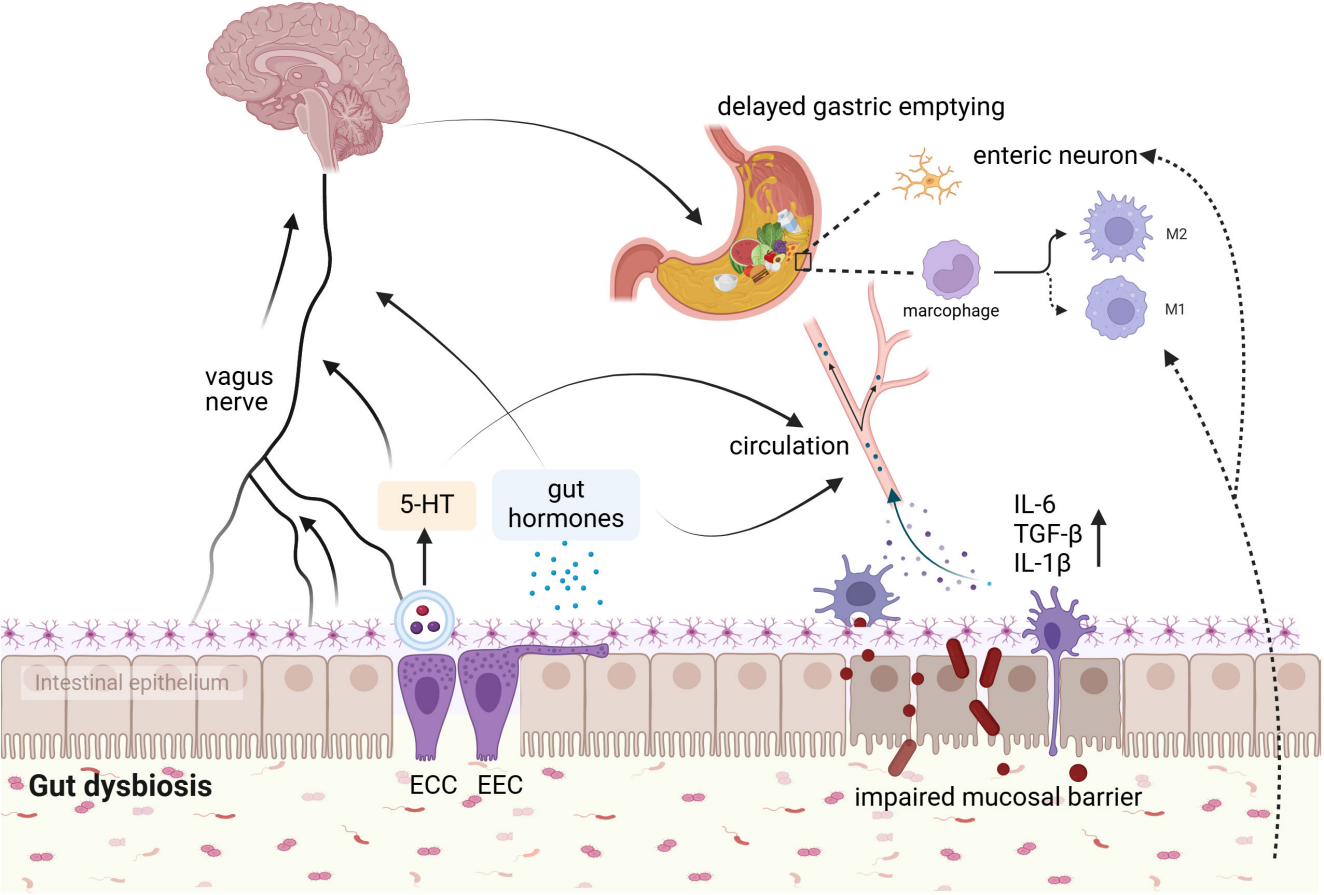
Potential mechanism of association between gut microbiota and postoperative DGE. The interaction between gut microbes and postoperative DGE may involve hormone secretion, 5-HT, gut mucosal barrier, enteric nerves systems, and muscularis macrophages. ECC, enterochromaffin cells; EEC, enteroendocrine cells (By BioRender.com).

As mentioned above, surgical procedures can lead to profound microbial alterations. Because of the vital role of gut microbiota in host physiology, it is reasonable to hypothesize that the imbalance within the microbiota may interact with other pathophysiological mechanisms to contribute to the initiation, development, or exacerbation of postoperative DGE. Recent research has demonstrated that 12α-hydroxylated bile acids have the ability to regulate gastric emptying in mice (Higuchi et al., 2020). Nonetheless, the specific mechanisms linking these microbiota alterations to gastric motility in the postoperative period remain largely unknown. Several speculative mechanisms are proposed below (**Figure 2**).

### 5.1 Intestinal microbiota and gastrointestinal hormones, 5-hydroxytryptamine

Brain peptides play a crucial role in the treatment of gastrointestinal motility disorders (Van den Houte et al., 2020; Mori et al., 2022). Abnormal levels of gastrointestinal hormones are one of the underlying mechanisms of postoperative gastrointestinal dysfunction (Wen et al., 2016), underscoring the importance of targeting these peptides for treatment. Falkén Y et al. demonstrated that a 3-hour intravenous infusion of ghrelin can accelerate gastric emptying rates in patients who have undergone colorectal surgery (Falkén et al., 2013).

Available evidence suggests that gut flora and their metabolites can modulate the release of gut hormones in many different ways (Neuman et al., 2015; Fukui et al., 2018; Sun et al., 2020), and the most well-studied are GLP-1 and peptide YY(PYY). Research has shown that serum levels of GLP-1 are significantly elevated in rats with gut microbiota dysbiosis induced by broad-spectrum antibiotics (Lai et al., 2024). GLP-1 and PYY are both predominantly produced by intestinal enteroendocrine L-cells, slow stomach emptying (Mori et al., 2022). Studies have shown that the microbiota can modulate the number and intestinal expression of the enteroendocrine L-cells population, thus regulating the secretion of GLP and PYY (Greiner and Bäckhed, 2016; Covasa et al., 2019). Further, research shows that *Enterococcus faecalis* Metalloprotease GelE is able to degrade GLP-1, which enhances insights into the specific host-microbiome intricate interactions (LeValley et al., 2020). Ghrelin stimulates the gastric emptying rate (Murray et al., 2005; Levin et al., 2006). A number of microbes and metabolites have been found seems to be positively or negatively associated with Ghrelin circulating levels (Schalla and Stengel, 2020). In addition to Ghrelin secretion, the intestinal microbiota can also affect Ghrelin signaling processes. It has been documented that the gut microbiome impacts growth hormone-releasing peptidergic signaling by influencing metabolites at the Ghrelin receptor level (Schalla and Stengel, 2020; Leeuwendaal et al., 2021). Research has shown that microbial metabolites such as butyrate, propionate, acetate, and lactate, as well as bacterial strain supernatants, influence the activation of a complex signal transduction cascade by growth hormone secretagogue receptor-1a in response to Ghrelin (Torres-Fuentes et al., 2019).

5-hydroxytryptamine (5-HT), also known as serotonin, plays a crucial role as a gastrointestinal neurotransmitter in regulating gastrointestinal motility. Agonists of the 5-HT_3_, 5-HT_4_, and 5-HT_4_ receptor have shown effectiveness in relieving symptoms in patients with gastroparesis, making them a significant pharmacological target of this condition (Abell et al., 2023; Naing et al., 2023). Approximately 90% of 5-HT in humans is synthesized by enterochromaffin cells (Banskota et al., 2019). In enterochromaffin cell-specific Tph1^CreERT2/+^ transgenic mouse, which enterochromaffin cells were depleted, showed a reduction in 5-HT levels, thus leading to delayed gastric emptying. Furthermore, patients with idiopathic gastroparesis demonstrated reduced 5-HT in gastric sinus EC cells and serum, which was inversely associated with delayed gastric emptying (Wei et al., 2021). This study confirms the significant role of 5-HT in the pathogenesis of gastroparesis for the first time (Wei et al., 2021). The gut microbiota and its metabolites can influence 5-HT synthesis and secretion directly or indirectly (Reigstad et al., 2015; Bhattarai et al., 2017; Yang et al., 2017). *Clostridium butyricum* could upregulate 5-HT levels in plasma, intestine by stimulating 5-HT secretion (Mandić et al., 2019). *Bifidobacterium dentis* increases acetate concentration in the mouse intestine, stimulates 5-HT secretion from enterochromaffin cells, and upregulates the expression of intestinal 5-HT receptors and 5-HT transporter proteins (Bonaz et al., 2018).

### 5.2 Intestinal microbiota and the central nervous system, enteric nervous system

It is well known that gastric motility is considerably innervated by the extrinsic neural signals originating from the dorsal vagal complex in the brainstem (Browning and Travagli, 2014). The pacemaker neurons in the dorsal motor nucleus of the vagus nerve are influenced by inputs from the nearby nucleus tractus solitarius and higher centers, including projections from the paraventricular nucleus of the hypothalamus (Wang et al., 2023). Studies suggest that disruptions in the innervation between the brain and gut, through the vagus nerve or enteric nervous system, are common in individuals with gastroparesis (Maksimaityte et al., 2021). Animal experiments have shown that abdominal surgery can trigger the release of corticotropin-releasing factor (CRF), which in turn slows gastric emptying. And the paraventricular nucleus of the hypothalamus and the dorsal vagal complex are key areas that respond to CRF-induced delays in gastric emptying (Wang et al., 2011).

The bidirectional intercommunication between the CNS, ANS, and ENS occurs through the brain-gut axis, with the vagus nerves and brain-gut peptides playing crucial roles in maintaining its normal function, while the gut microbiota acts as key mediators of brain-gut interactions, closely influencing gastrointestinal motility regulation. The gut microbiota regulate CNS activities through neural, immune, and endocrine pathways (Cryan et al., 2019). On the one hand, certain metabolites produced by gut microbes can enter the bloodstream and travel to the associated brain regions to influence CNS functioning and actions (Sharon et al., 2016). Additionally, it has been suggested that the afferent branch of the vagus nerve can detect microbial signals that manifest as bacterial metabolites (Margolis et al., 2021). These pieces of information are transmitted upward via afferent nerves to the CNS, which receives and integrates a range of relevant information from both internal and external environments. Subsequently, the CNS sends regulatory signals through the autonomic nerve and neuroendocrine system to the gastrointestinal plexus or directly to target cells within the gastrointestinal tract (Bonaz et al., 2018; Wang et al., 2020). Surprisingly, latest research demonstrated that hypothalamic neurons can detect changes in gut microbiota structure (Gabanyi et al., 2022). Together, multiple potential pathways exit, both direct and indirect, through which altered gut microbiota can modulate the vague nerve signals (Fülling et al., 2019). However, there is no relevant research has been performed to explore whether the changes in microbiota influence the brain actions involved in gastric emptying.

Normal enteric nerve fibers play a role in gastric emptying through motor pathways that involve inhibitory and excitatory neurotransmitters. Study have shown that transgenic mice with disrupted intestinal glial cells exhibit delayed gastric emptying compared to normal mice (P<0.05) (Aubé et al., 2006). Evidence suggests that gut microbes are essential for promoting the survival and maintenance of enteric neurons. In germ free mice, there was a reduction in both nitrergic and total neurons in the distal ileum compared to control mice (Anitha et al., 2012). Nitric oxide released by enteric nerves plays a key role in regulating gastric emptying by controlling pyloric sphincter relaxation (Idrizaj et al., 2021). Physiologically, gastric emptying requires functional GLP-1 receptors and intestinal neuronal nitric oxide synthase, which need to function in an intestinal environment with beneficial microflora (Grasset et al., 2017). Gut microbiota dysbiosis can reduce GLP-1R and neuronal nitric oxide synthase expression in the ENS, leading to inhibited gastric emptying by blocking GLP-1-induced nitric oxide production through a pattern recognition receptor-dependent mechanism (Yamane and Inagaki, 2018).

### 5.3 Intestinal microbiota and the intestinal mucosal barrier

The intestinal mucosal barrier is comprised of biological, mechanical, immune, and chemical barriers (Yang et al., 2021). Normally, the intestinal mucosal barrier is able to segregate the intestinal luminal contents, prevent the invasion of pathogenic antigens into the submucosal tissues and circulatory system. Notably, a present clinical study reported that patients with DGE have higher serum levels of pro-inflammatory cytokines, such as interleukin-1β, and tumor necrosis factor α, in conjunction with damaged intestinal mucosal barrier integrity, compared to patients with normal gastric emptying (Greis et al., 2017). Martinez et al. found a decrease in zonulin, a protein considered a marker of the integrity of the intestinal mucosal barrier, was associated with DGE in surgical critically ill pediatric patients (Martinez et al., 2020). Moreover, a recent study reported a novel regulatory mechanism of gastric emptying in humans, that the increasing concentrations of interleukin-6 delays gastric emptying in human (Lehrskov et al., 2018).

The intestinal microbiota acts as a biological barrier to the intestinal mucosa. Abnormal intestinal flora can impair the structure and function of the intestinal mucosal barrier through a variety of mechanisms, such as regulating the intestinal mucus barrier function, and the expression of tight junction proteins (Allam-Ndoul et al., 2020; Paone and Cani, 2020). Besides, it is reported that a decrease in short-chain fatty acids can result in impaired intestinal mucosal barrier and bacterial translocation (Schreiber et al., 2024). Toll-like receptors are a group of pattern-recognition receptors that detect microbial components and trigger the production of inflammatory cytokines. The composition of the intestinal flora can affect the function of the intestinal mucosal barrier by altering the toll-like receptors signaling pathway. An animal study reported that surgery can shift gut microbiological composition, and these microbiological changes were shown to exacerbate the disruption of the intestinal barrier in aged mice (Pan et al., 2023). Increased intestinal permeability allows the translocation of toxic metabolites, pathogens, or other antigens into the bloodstream and thus increase circulating levels of molecules that contribute to the activation of inflammatory pathways and the release of pro-inflammatory cytokines, such as interleukin-6, tumor necrosis factor α, etc (König et al., 2016). It has been reported that some pro-inflammatory cytokines or mediators may decrease gastrointestinal motility (Docsa et al., 2022). Okdahl et al. found that prolonged gastric emptying in individuals with diabetes was associated with elevated serum levels of interleukin-8, and the subjective cardinal symptoms of gastroparesis were negatively correlated with interleukin-6 levels (Okdahl et al., 2023).

### 5.4 Intestinal microbiota and macrophages

Muscularis macrophages carry out vital functions in the regulation of gastrointestinal motility (Muller et al., 2014; Chikkamenahalli et al., 2024), which can be classified as pro-inflammatory M1 types and anti-inflammatory M2 types. Studies have established that the quantity and phenotype of macrophages present in the gastrointestinal tract are important factors in the development of gastroparesis (Cipriani et al., 2016, 2018). Specifically, Grover et al. found a heightened immunoreactivity of CD68 in patients suffering from gastroparesis through full thickness biopsies, indicating an increased presence of M1 macrophages in these patients (Grover et al., 2011). Furthermore, Grover and colleagues noted a deficiency of CD206, a marker for M2 type macrophages, in patients with diabetic gastroparesis and idiopathic gastroparesis (Grover et al., 2017). Abdominal surgery activates M1 type macrophages in the gastrointestinal plexus of rats and increases the expression of pro-inflammatory cytokines; additionally, gastric emptying is significantly and negatively correlated with MHCII/CD206^+-^ (M1) macrophages in the gastrointestinal plexus (Yuan and Taché, 2017).

Several studies have identified that the intestinal microbiota regulates gastrointestinal peristalsis partly through influencing the interactions between muscularis macrophages and enteric neurons (Muller et al., 2014). Becker et al. demonstrated that alterations in gut microbiota contribute to changes in macrophage phenotype (Becker et al., 2018). Yang et al. conducted a study to investigate the impact of gut microbiota on the relationship between gastrointestinal motility, 5-HT expression, and macrophage abundance. They found a notable rise in the numbers of muscularis MR-positive macrophages in the upper gastrointestinal and colon of germ-free mice that received fecal transplantation compared with germ-free mice without transplantation (Yang et al., 2017).

Although surgery may directly cause impaired gastric motility through factors such as vagal injury, anatomical changes, etc., it should not be excluded that DGE could also be partially affected by microbiome alterations resulting from surgery. While intriguing, it should be pointed out that this highly theoretical hypothetical scenario nevertheless warrants further clinical and experimental studies to either support or invalidate it of action.

## 6 Possible therapeutic strategies based on microbiological modulation of postoperative gastric motility

Current therapeutic options for managing gut dysbiosis include probiotics, prebiotics, synbiotics, dietary interventions, fecal microbiota transplantation (FMT), and others. However, research on their application in DGE is limited. Several studies, as discussed below, have shown the efficacy of this approach in improving digestive clinical symptoms (**Table 2**).

**Table 2:** Potential intervention for postoperative DGE.

Interventions	Patients	Selected results of interventions	Reference
*Clostridium butyricum*	gastric cancer patients after gastrectomy	inhibited inflammatory response; restore intestinal microbiota eubiosis; reduce the incidence of postoperative gastrointentional adverse reactions (including abdominal pain, bloating, diarrhea, constipation)	Cao et al., 2022
probiotic compounds (including *Lactobacillus plantarum* MH-301, *L. rhamnosus* LGG-18, *L. acidophilus*, *Bifidobacterium animalis subsp.lactis* LPL-RH)	gastric cancer patients after radical distal gastrectomy	reduce the inflammatory response; shorter the postoperative gastrointestinal recovery time	Xiong et al., 2024
probiotic (including *Lactobacillus plantarum*, *Lactobacillus acidophilus*, *Bifido-bacterium longum*)	colorectal cancer patients undergoing colorectomy	improve the integrity of gut mucosal barrier and the postoperative recovery of peristalsis; shorten the first defecation time	Liu et al., 2011
autoprobiotics	colorectal cancer after surgery	reduce dyspeptic symptoms	Ermolenko et al., 2024

In recent years, probiotics have gained attention for their role in postoperative recovery (Krezalek and Alverdy, 2016; Kotzampassi, 2022; Xiong et al., 2024). Evidence suggests that oral administration of *Clostridium butyrate* after gastric cancer surgery can help restore postoperative intestinal flora, improve inflammation and immunity, and thus reduce postoperative complications (Cao et al., 2022). A clinical trial was conducted to investigate the effect of probiotic compounds on postoperative recovery in patients with distal gastric cancer. The patients were randomly assigned to a probiotic group or a placebo group. The results showed that patients in the probiotic group exhibited an earlier recovery of gastrointestinal function than those in the placebo group (Xiong et al., 2024). Furthermore, a meta-analysis supported the benefits of perioperative probiotic or synbiotic supplementation in facilitating gastrointestinal recovery in postoperative patients with gastrointestinal malignancies. The supplementation was linked to a reduction in the number of days to the first solid and liquid diet, a decrease in postoperative hospital stay, and a lower occurrence of postoperative abdominal distension and intestinal obstruction (Tang et al., 2022). In patients with colorectal cancer undergoing colorectomy, the perioperative probiotic treatment benefits the fecal microbiota, improves the integrity of the gut mucosal barrier, and shortens the first defecation, but there are no statistically significant differences in the fluid and solid intake times (Liu et al., 2011). A recent study observed that the administration of autoprobiotics in the early postoperative period can decrease dyspeptic complaints after surgery for colorectal cancer (Ermolenko et al., 2024). This may involve positive alterations in the gut microbiota.

These findings suggest that probiotics regulating the intestinal flora balance appear to be a potentially promising therapeutic approach or an add-on therapy to an approved approach for postoperative DGE. However, future large-scale trials are necessary to verify the effectiveness of restoring gut flora imbalance in improving postoperative gastrointestinal dysfunction.

Furthermore, recent research has indicated the possibility of dietary interventions to restore the balance of the gut microbiota in disease. It has been proposed that modifying the gut microbiota through dietary interventions in patients with colorectal cancer may be an effective approach to improving perioperative dysbiosis and postoperative outcomes (Martínez-Montoro et al., 2022).

FMT, a method to reconstruct gut microbiota that involves the transfer of fecal material from a healthy donor to another individual to treat disease, shows a promise in intervening in gastrointestinal motility disorders (Antushevich, 2020). However, the application of FMT should be approached with caution and a comprehensive understanding of its potential benefits and risks.

## 7 Prospects

The postoperative restoration of gastrointestinal motility is a key issue in facilitating rapid perioperative recovery. It is possible that dysbiosis of the gut flora may contribute to the development of DGE. However, this field is still at the exploratory stage, and it has not yet been demonstrated whether these changes are responsible for the postoperative DGE outcomes and, if so, to what extent. It would be beneficial for future studies to focus on investigating the microbiota changes associated with postoperative impaired gastric motility in large patient cohorts. Furthermore, it would be advantageous to validate these findings through fecal transplantation experiments to determine the causal relationship. This would help identify specific bacterial taxa that are associated with postoperative gastroparesis. Additionally, mucosa-associated microbiomes seem to exert a more pathogenic influence compared to fecal microbes. A comprehensive understanding of the interactions between microbiota and DGE may be helpful in designing novel non-invasive diagnostic tools utilizing DGE-specific microbiota profiles and metabolites, as well as strategies for the management and prevention of this gastrointestinal disorder. Currently, evidence suggests that preoperative gut flora may serve as a predictive factor for postoperative intestinal paralysis (Jin et al., 2020), inspiring us to investigate the predictive role of altered preoperative gut flora in DGE.
